# **Development of a Rapid Derivative Spectrophotometric Method for Simultaneous Determination of Acetaminophen, Diphenhydramine and Pseudoephedrine in Tablets**

**Published:** 2015

**Authors:** Effat Souri, Aghil Rahimi, Nazanin Shabani Ravari, Maliheh Barazandeh Tehrani

**Affiliations:** *Department of Medicinal Chemistry, Faculty of Pharmacy and Pharmaceutical Research Center, Tehran University of Medical Sciences, Tehran, Iran.*

**Keywords:** Acetaminophen, Diphenhydramine hydrochloride, Pseudoephedrine hydrochloride, Derivative spectrophotometry

## Abstract

A mixture of acetaminophen, diphenhydramine hydrochloride and pseudoephedrine hydrochloride is used for the symptomatic treatment of common cold. In this study, a derivative spectrophotometric method based on zero-crossing technique was proposed for simultaneous determination of acetaminophen, diphenhydramine hydrochloride and pseudoephedrine hydrochloride. Determination of these drugs was performed using the ^1^D value of acetaminophen at 281.5 nm, ^2^D value of diphenhydramine hydrochloride at 226.0 nm and ^4^D value of pseudoephedrine hydrochloride at 218.0 nm.

The analysis method was linear over the range of 5-50, 0.25-4, and 0.5-5 µg/mL for acetaminophen, diphenhydramine hydrochloride and pseudoephedrine hydrochloride, respectively. The within-day and between-day CV and error values for all three compounds were within an acceptable range (CV<2.2% and error<3%). The developed method was used for simultaneous determination of these drugs in pharmaceutical dosage forms and no interference from excipients was observed.

## Introduction

**Figure 1:**
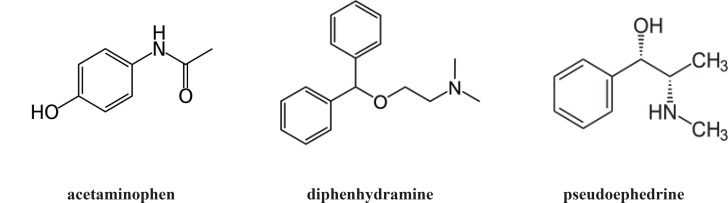
Chemical structure of acetaminophen, diphenhydramine, and pseudoephedrine.

Acetaminophen, chemically known as N-(4-hydroxyphenyl) acetamide, is an antipyretic-analgesic agent used in different pharmaceutical dosage forms (1). Diphenhydramine hydrochloride, 2-(diphenylmethoxy)-N, N-dimethylethanamine hydrochloride, is a reversible H_1_ antagonist which is used in the symptomatic treatment of allergic diseases (1). Pseudoephedrine hydrochloride, (1S, 2S)-2-methylamino-1-phenylpropan-1-ol hydrochloride, is a sympathomimetic agent and is effective for the relief of nasal congestion (1). Chemical structures are shown in Figure 1. A combination of an analgesic, antihistamine and decongestant is commonly used to treat the symptoms of common cold. 

A survey of literature showed that there are several spectrophotometric (2, 3), HPLC (4-11) or LC-MS-MS (12-14) methods for the determination of these drugs alone or in combination dosage forms. The assay method for combination preparations of these drugs cited in the USP, is based on a reversed-phase HPLC using a CN column and a mixture of phosphate buffer, acetonitrile and triethylamine as mobile phase with a relatively long chromatographic analysis time. Also another reversed-phase HPLC method has been reported for the simultaneous determination of acetaminophen, pseudoephedrine hydrochloride, diphenhydramine hydrochloride and dextromethorphan hydrobromide in dosage forms (15). Although the reported HPLC methods are sensitive and offer a high degree of specificity, they are relatively expensive.

There has been no spectrophotometric method reported for simultaneous determination of these drugs. The development and evaluation of spectrophotometric methods can reduce the time and cost of the analysis. Due to the spectral overlap of these drugs, they could not be determined by classical spectrophotometric methods. Spectrophotometric analysis based on chemometrics is reported to solve this problem (2, 3). Derivative spectrophotometric methods could also be used for spectral resolution of multi-component mixtures and simultaneous determination of compounds with overlapped spectra. Derivative spectrophotometric methods are very simple, rapid, and reliable techniques for simultaneous determination of compounds with overlapped spectra and spectrophotometric methods have been received increasing attention (16-26). These methods could be used without any pretreatment procedures and tedious sample preparations. The goal of this study was to develop a practical, reliable and fast derivative spectrophotometric method for the simultaneous determination of acetaminophen, diphenhydramine hydrochloride and pseudoephedrine hydrochloride in a multicomponent formulation.

## Experimental 

*Instrumentation*

 Shimadzu double-beam UV-visible spectrophotometer (Model 160, Kyoto, Japan) was used for spectrophotomertic determinations. The zero order and derivative spectra were recorded in the range of 200-300 nm.

*Chemicals*

Acetaminophen bulk powder was prepared from Temad Co., Mashhad, Iran (Batch No. Ac 903304). Diphenhydramine hydrochloride USP was from Ipca Laboratories Limited, Mambai, India (Batch No. 0001F1RJ) and kindly provided by Hejrat Distribution Co., Tehran, Iran. Pseudoephedrine hydrochloride was from Malladi Drugs & Pharmaceuticals Limited, Chennai, India (Batch No. 5002311) and kindly provided by Dr Abidi Pharmaceutical Laboratory, Tehran, Iran. Coldax® tablets (500 mg acetaminophen, 25 mg diphenhydramine hydrochloride and 30 mg pseudoephedrine hydrochloride) were from Dr Abidi Pharmaceutical Laboratory, Tehran, Iran (Batch No. 57 9 90) and obtained from a local pharmacy.

*Standard solutions*

Standard solutions of 100 µg/mL of acetaminophen, 10 µg/mL of diphenhydramine hydrochloride and 10 µg/mL of pseudoephedrine hydrochloride were prepared in 0.1 M HCl.

To prepare the calibration solutions of acetaminophen in the presence of other drugs, suitable amounts of standard solutions of acetaminophen (100 µg/mL) ranging form 0.5 to 4 mL were transferred into separate 10 mL volumetric flasks to produce concentrations of 5, 10, 15, 20, 25, 30, 35, and 40 µg/mL. 1.5 mL of diphenhydramine hydrochloride solution (10 µg/mL) and 1.5 ml of pseudoephedrine hydrochloride (10 µg/mL) solution were added to each flask. The solutions diluted to the mark with 0.1M HCl.

The same procedure was performed to prepare the calibration solutions of diphenhydramine hydrochloride at 0.25, 0.5, 1, 1.5, 2, 2.5, 3, 3.5, and 4 µg/mL in the presence of constant concentration of acetaminophen (25 µg/mL) and pseudoephedrine hydrochloride (1.5 µg/mL).

The calibration solutions of pseudoephedrine hydrochloride at 0.5, 1, 1.5, 2, 2.5, 3, 3.5, 4, 4.5, and 5 µg/mL in the presence of acetaminophen (25 µg/mL) and diphenhydramine hydrochloride (1.5 µg/mL) were also prepared by the same procedure.

*Spectrophotometric measurement*

Standard solutions of acetaminophen (100 µg/mL), diphenhydramine hydrochloride (10 µg/mL) and pseudoephedrine hydrochloride (10 µg/mL) was separately subjected to zero order spectrophotometric measurements using 0.1 M HCl as blank in the range of 200-300 nm. The first order to fourth order derivative spectra was obtained in the same wavelength range and different Δλ values.

The ^1^D (Δλ=28.0) values for acetaminophen at 281.5 nm (zero-crossing of pseudoephedrine and diphenhydramine), ^2^D (Δλ=31.5) values for diphenhydramine at 226.0 nm (zero-crossing of acetaminophen and pseudoephedrine), and ^4^D (Δλ=27.0) values for pseudoephedrine at 218.0 nm (zero-crossing of acetaminophen and diphenhydramine) were used for spectrophotometric determinations.

*Validation of the method*

To evaluate the linearity of the proposed method, six series of calibration solutions of each component in the presence of other drugs were determined. The ^1^D values at 281.5 nm,^ 2^D values at 226.0 nm and ^4^D values at 218.0 nm were measured for acetaminophen, diphenhydramine hydrochloride and pseudoephedrine hydrochloride, respectively and plotted against the analyte concentration. The statistical analysis for the slope and intercept was performed.

For the evaluation of the accuracy and precision of the developed method, synthetic mixtures for each component at three different concentrations in the calibration range were prepared. These solutions were analyzed according to the above mentioned method using their corresponding calibration curves. This procedure was repeated three times in one day and three consecutive days.

*Application of the method*

Ten tablets of Coldax® were weighed and finely powdered. An amount of the resulted powder equivalent to one fourth of one tablet was quantitatively transferred to a 100 mL volumetric flask and 70 mL of 0.1 M HCl was added. After sonication for 15 min, the flask was completed to volume by 0.1 M HCl. The solution was filtered through a 0.45 µm membrane filter (Millipore) and 3 mL of the filtrate were transferred to a 100 mL volumetric flask and diluted with 0.1 M HCl. The concentrations of the active ingredients were determined. The assay method was also performed according to the standard USP method. The content of each drug was calculated by comparison with an appropriate standard solution of the drugs at appropriate concentration.

## Results and Discussion

*Spectrophotometric measurements*

**Figure 2:**
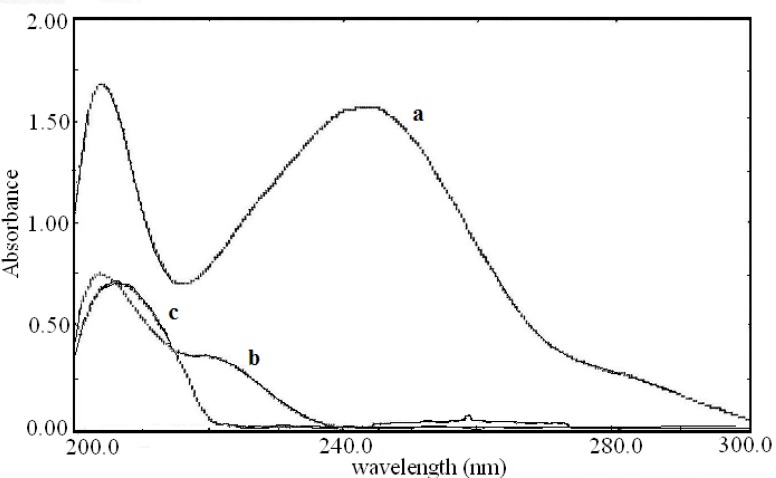
Zero order spectra of (a) acetaminophen (20 μg/mL), (b) diphenhydramine hydrochloride (10 μg/mL) and (c) pseudoephedrine hydrochloride (7 μg/mL).

The zero order absorption spectra of acetaminophen, diphenhydramine hydrochloride and pseudoephedrine hydrochloride obtained in 0.1 M HCl solution in the presence of 0.1 M HCl as blank are shown in Figure 2. As direct simultaneous measurement of these drugs is not possible, the derivative spectra were examined. The derivative spectra of all three compounds were obtained at different orders and varied Δλ values to select the more suitable order of the derivative for measurements. 

**Figure 3:**
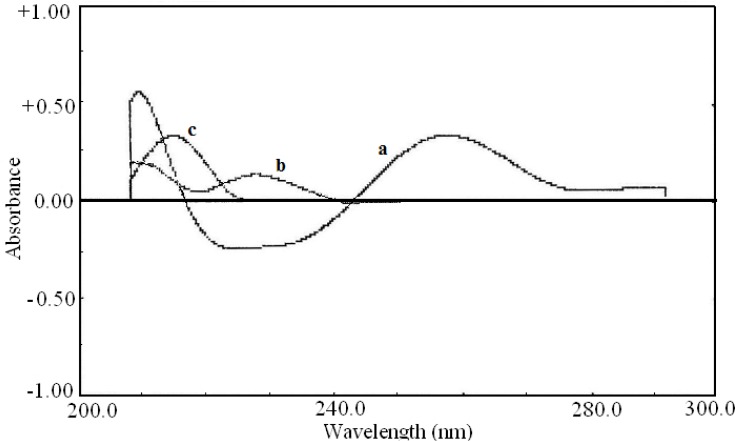
First order derivative spectra of (a) acetaminophen (20 μg/mL), (b) diphenhydramine hydrochloride (10 μg/mL) and (c) pseudoephedrine hydrochloride (7 μg/mL).

The first order derivative spectra traced with Δλ= 28.0 nm are shown in Figure 3. The zero-crossing point for pseudoephedrine hydrochloride and diphenhydramine hydrochloride at 281.5 nm could be used for determination of acetaminophen in the presence of other drugs (Figure 3).

**Figure 4:**
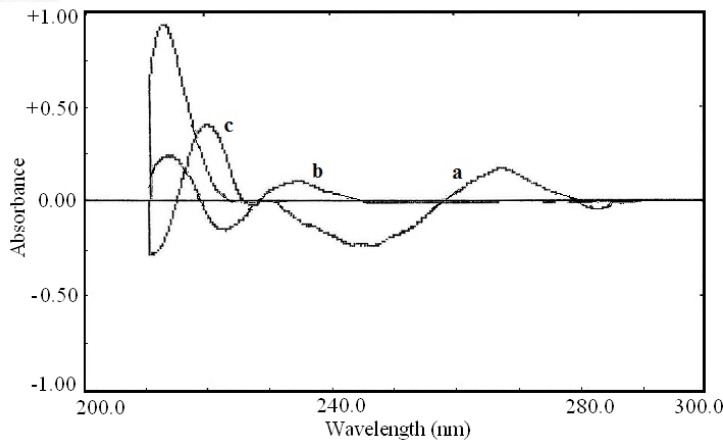
Second order derivative spectra of (a) acetaminophen (20  g/mL), (b) diphenhydramine hydrochloride (10  g/mL) and (c) pseudoephedrine hydrochloride (7  g/mL).

**Figure 5:**
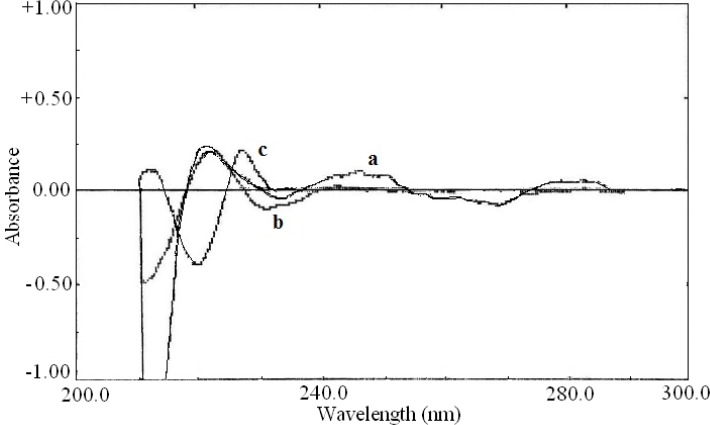
Fourth order derivative spectra of (a) acetaminophen (20 μg/mL), (b) diphenhydramine hydrochloride (10 μg/mL) and (c) pseudoephedrine hydrochloride (7 μg/mL).

The second order derivative and fourth order derivative spectra were also showed suitable wavelength for determination of other drugs. The zero-crossing point for acetaminophen and pseudoephedrine hydrochloride at 226.0 nm in the second order derivative spectra (Figure 4) could be used for the determination of diphenhydramine hydrochloride. Also the zero-crossing point for acetaminophen and diphenhydramine hydrochloride at 218.0 nm in the fourth order derivative spectra (Figure 5) was suitable for quantification of pseudoephedrine hydrochloride. These selected wavelengths, showed the best linear response to the analyte concentration, which was not affected by the concentration of other components.

*Linearity*

The calibration solutions of each compound in the presence of constant concentration of the two other compounds were determined at the above mentioned wavelengths using their corresponding derivative spectra. The calibration curves were constructed and the statistical data obtained for six calibration curves were calculated and presented in Table 1. 

The limit of quantification (LOQ) and the limit of detection (LOD) were calculated according to the following equations (27) and are shown in Table 1.

LOQ= 10/s and LOD=3.3/s 

Where is the standard deviation of intercept and s is the slope of the calibration graph.

**Table 1:** Statistical data of calibration curves of acetaminophen, diphenhydramine hydrochloride and pseudoephedrine hydrochloride.

**Parameters**	**Acetaminophen** a	**Diphenhyramine** b	**Pseudoephedrine** c
	^1^ **D**_281.5_ ** (Δλ=** **28.0** **) **	^2^ **D**_226.0_ ** (Δλ=** **31.5** **) **	^4^ **D**_218.0_ ** (Δλ=** **27.0** **) **
**Linearity range**	5-40 g/mL	0.25-4 g/mL	0.5-5 g/mL
**Regression equation**	Y=0.00295X-0.0024	Y=0.01193 X+0.01275	Y=0.03598 X+0.0603
**SD of slope**	5.510^-5^	0.00036	0.00081
**RSD of slope (%)**	1.86	2.98	2.25
**CI ** d ** of slope**	4.410^-5^	0.00029	0.00065
**SD of intercept**	0.00047	0.00070	0.0031
**CI ** d **of intercept**	0.00037	0.00056	0.0025
**Correlation coefficient**	0.999	0.997	0.996
**LOQ**	1.59	0.86	0.59
**LOD**	0.53	0.28	0.19

a In the presence of diphenhydramine (1.5 g/mL) and pseudoephedrine (1.5 g/mL)

b In the presence of acetaminophen (25 g/mL) and pseudoephedrine (1.5 g/mL)

c In the presence of acetaminophen (25 g/mL) and diphenhydramine (1.5 g/mL)

d CI: Confidence Interval (P=0.05)

*Precision and accuracy*

Synthetic mixtures of acetaminophen, diphenhydramine hydrochloride and pseudoephedrine hydrochloride were determined to find out the within-day and between-day precision and accuracy. The summary of the results are shown in Table 2 and satisfactory results were obtained over the stated calibration range. 

**Table 2:** Accuracy and precision data for simultaneous determination of acetaminophen^a^, diphenhydramine hydrochloride^b^ and pseudoephedrine hydrochloride^c^ (3 sets for 3 days) by derivative spectrophotometry.

**Added (g/mL)**	**Within-day (n = 3)**			**Between-day (n = 9)**		
	**Found** **(g/mL)**	**CV (%)**	**Error (%)**	**Found** **(g/mL)**	**CV (%)**	**Error (%)**
**Acetaminophen** ^1^ **D**_281.5_ ** (Δλ=** **28.0** **)**						
**5.00** **20.00** **40.00**	5.08± 0.08 19.78± 0.05 39.92± 0.20	1.57 0.25 0.50	1.60 -1.10 -0.20	5.03± 0.11 19.84± 0.32 40.07±0.44	2.19 1.61 1.10	0.60 -0.80 0.18
**Diphenhydramine** ^2^ **D**_226.0_ ** (Δλ=** **31.5** **)**						
**0.50** **2.00** **4.00**	0.50±0.01 1.99± 0.04 3.94± 0.03	2.00 2.01 0.76	0.00 -0.50 -1.50	0.50± 0.01 2.01± 0.03 3.97± 0.05	2.00 1.49 1.26	0.00 0.50 -0.75
**Pseudoephedrine** ^4^ **D**_218.0_ ** (Δλ=** **27.0** **)**						
**1.00** **3.00** **5.00**	0.97±0.02 3.02± 0.05 4.95± 0.08	2.06 1.66 1.62	-3.00 0.07 -0.10	0.98± 0.02 3.01± 0.05 4.97± 0.06	2.04 1.66 1.21	-2.00 0.33 -0.60

a in the presence of diphenhydramine (1.5 g/mL) and pseudoephedrine (1.5 g/mL)

b in the presence of acetaminophen (25 g/mL) and pseudoephedrine (1.5 g/mL)

c in the presence of acetaminophen (25 g/mL) and diphenhyramine (1.5 g/mL)

*Relative recovery*

The relative recovery of each component was checked by using standard addition method. Standard concentrations of pure drugs were added to tablet solution and the relative recovery was calculated. The recoveries were reported to be 101.5±1.4%, 99.3±1.3% and 100.1±0.9% for acetaminophen, diphenhydramine hydrochloride and pseudoephedrine hydrochloride, respectively which shows no significant interference from the excipients.

*Application of the method*

The results of the analysis of Coldax® tablets using the proposed method alongside with the reference USP method are shown in Table 3. The results are in good agreement with the declared amount of the components on the label. Student's paired t-test and the Variance ratio F-test results showed no significant difference between the proposed method and the reference method.

**Table 3:** Comparison of the developed method with the reference method for the determination of Coldax® tablets.

**Compound**	**Label claimed(mg)**	**Found(mean ± sd)**		**Statistical Tests** *
		**Proposed method**	**HPLC method**	
**Acetaminophen**	500.00	507.25±7.20	507.49±4.48	t = 0.960 F = 0.553
**Diphenhydramine**	25.00	25.00±0.81	25.08±0.40	t = 0.915 F = 0.391
**Pseudoephedrine**	30.00	29.92±0.26	30.03±0.57	t = 0.838 F = 0.354

* Theoretical values of t and F at p = 0.05 are 2.776 and 19.00 respectively.

## Conclusion

A derivative spectrophotometric method has been developed for the simultaneous determination of acetaminophen, diphenhydramine hydrochloride and pseudoephedrine hydrochloride in pharmaceutical dosage forms. The developed method is simple, accurate, cost effective, and practical for routine quality control analysis. Furthermore, a simple and rapid sample preparation is needed when applied to the analysis of pharmaceutical dosage forms.
